# Annotations

**Published:** 1908-04-18

**Authors:** 


### ANNOTATIONS.

### The Gyroscope at Sea.

At various times lately the public have been taken into the confidence of the engineering profession with regard to an invention which is to abolish sea-sickness. It now appears that a German ship is actually being fitted with a gyroscopic fly-wheel by which it is expected that rolling will be entirely obviated in any sea that is likely to be encountered. The diameter of this fly-wheel is to be some 63 inches, and is to revolve 1,800 times in a minute: this tremendous engine is designed for the necessities of a steamer of but 900 tons burden. The medical profession has always been to all intents helpless in connection with sea-sickness, the pathological mechanicism of which is even now not understood; it is good news, therefore, that one of the causes of this malady will henceforth be largely under control. We say one of the causes, because we do not gather that any gyroscope or other invention will diminish pitching, a process which to many is even more prejudicial than rolling. Even could this movement be abolished also, it is probable that a few would continue to experience sickness at sea; for the sight of the waves, or even, in rare cases, of a perfectly smooth sea, is sufficient sometimes to bring on the symptoms. But to the much larger class of moderate sailors, who can by various means, such as previous purgation, postural treatment, hypnotic drugs, and so forth, avoid the malady as long as the ship's oscillations are moderate in degree, the new invention, if it fulfils the claims made on its behalf, will prove a great boon. To the medical profession it will be especially interesting, as it may help to clear up some of the mystery attaching to that extraordinary disease, one of the rare affections to which infants and the aged are much more immune than adults in the prime of life.

### A "Doctor's Dilemma."

The obligation of a medical man to respect the confidences of his patients is one which now and then leads him into most difficult positions. A case in point has recently been the subject of litigation in France, and it is in all essentials similar to one which was decided in this country three or four years ago. In the former instance a club doctor was summoned to attend a member whom he found suffering from a contagious disease of a nature to disqualify him from the receipt of sick-pay under the club rules. He notified the diagnosis to the club authorities, and has in consequence been fined 500 francs and costs. In the latter, if we rightly remember, a medical man was paid by a golf club to treat one of the attendants at the club-house, and he conceived it his duty to the members to advise the patient's discharge, for which he had subsequently to pay heavy damages. In neither case was the accuracy of the diagnosis successfully contested. Now in each case the physician blundered: in the first, as we conceive, he should merely have refused his certificate; in the second he should have refused to continue in attendance unless the patient gave leave for the communication, and thus have left the club committee to draw their own conclusions. But at the same time it must be evident that when a medical man is employed and paid to safeguard the interests of a corporation and of its members individually, he would be guilty of a very gross betrayal of trust if he neglected so to do. The State admits that the physician's citizenship overrides his duty to his patient in the matters of notifiable diseases and of births. On the same principle it seems a hardship that the performance of a public duty under circumstances similar to those detailed should incur a heavy penalty. The only escape from dilemmas such as these is to proceed with the very greatest caution, and to ask for the patient's consent before witnesses or in writing.

### Iron and the Teeth.

Iron has been known from time immemorial to injure the teeth by discolouring them. There is not, however, a great deal of scientific knowledge as to why this should be so, and it is of some interest to read of the experiments made by Morgenstern, of Strassburg, upon the subject. This observer shows conclusively that it is not the iron that is secreted by the saliva that affects the teeth in this way, but only the actual medicaments that are being taken. He took sections of teeth and steeped them in all manner of different iron solutions, and he came to the conclusion that the only pharmacopœial preparation that did not stain them was ferrum redactum, though one or two proprietary remedies were also non-staining. All fluid medicaments containing iron should be taken through a glass tube, unless a tooth-brush is used regularly after each dose.

### Medical Superintendents' Salaries.

At a recent meeting of the managers of the Metropolitan Asylum District the application of the medical superintendents in the hospitals' service for an increase of their remuneration was considered. The salary paid at present to a medical superintendent begins at £400 per annum, and rises annually by £25 to a maximum of £700 per annum, with certain emoluments. The medical superintendents have had greatly increased responsibility and work lately, and during the recent fever epidemic, unparalleled in the history of the Board, they loyally co-operated with the committee, with the result that not one patient was refused admission. It was resolved that from March 25 next the salary shall begin at £500 per annum, rising by four annual increments of £25 to £600; and thence by two further annual increments of £50 to £700 per annum; and that after the expiration of ten completed years of service the salary shall be increased from £700 by £50 annually to £800 per annum, with emoluments. It was further resolved that, subject to the assent of the Local Government Board, the salary of each of the nine senior medical superintendents be increased to £800 per annum from March 26, 1908.

